# Perturbation of lipids and glucose metabolism associated with previous 2,4-D exposure: a cross-sectional study of NHANES III data, 1988-1994

**DOI:** 10.1186/1476-069X-9-11

**Published:** 2010-02-26

**Authors:** Dina M Schreinemachers

**Affiliations:** 1 Epidemiology Branch, Environmental Public Health Division, National Health and Environmental Effects Research Laboratory, Office of Research and Development, US Environmental Protection Agency, 109 TW Alexander Drive, Research Triangle Park, NC 27711, USA

## Abstract

**Background:**

Results from previous population studies showed that mortality rates from acute myocardial infarction and type-2 diabetes during the 1980s and 1990s in rural, agricultural counties of Minnesota, Montana, North and South Dakota, were higher in counties with a higher level of spring wheat farming than in counties with lower levels of this crop. Spring wheat, one of the major field crops in these four states, was treated for 85% or more of its acreage with chlorophenoxy herbicides. In the current study NHANES III data were reviewed for associations of 2,4-dichlorophenoxy acetic acid (2,4-D) exposure, one of the most frequently used chlorophenoxy herbicides, with risk factors that are linked to the pathogenesis of acute myocardial infarction and type-2 diabetes, such as dyslipidemia and impaired glucose metabolism.

**Methods:**

To investigate the toxicity pattern of chlorophenoxy herbicides, effects of a previous 2,4-D exposure were assessed by comparing levels of lipids, glucose metabolism, and thyroid stimulating hormone in healthy adult NHANES III subjects with urinary 2,4-D above and below the level of detection, using linear regression analysis. The analyses were conducted for all available subjects and for two susceptible subpopulations characterized by high glycosylated hemoglobin (upper 50^th ^percentile) and low thyroxine (lower 50^th ^percentile).

**Results:**

Presence of urinary 2,4-D was associated with a decrease of HDL levels: 8.6% in the unadjusted data (p-value = 0.006), 4.8% in the adjusted data (p-value = 0.08), and 9% in the adjusted data for the susceptible subpopulation with low thyroxine (p-value = 0.02). An effect modification of the inverse triglycerides-HDL relation was observed in association with 2,4-D. Among subjects with low HDL, urinary 2,4-D was associated with increased levels of triglycerides, insulin, C-peptide, and thyroid stimulating hormone, especially in the susceptible subpopulations. In contrast, subjects with high HDL did not experience adverse 2,4-D associated effects.

**Conclusions:**

The results indicate that exposure to 2,4-D was associated with changes in biomarkers that, based on the published literature, have been linked to risk factors for acute myocardial infarction and type-2 diabetes.

## Background

Chlorophenoxy herbicides, such as 2,4-dichlorophenoxy acetic acid (2,4-D) and 4-methyl-2-chlorophenoxyacetic acid (MCPA) have been used since World War II for weed control [1]. Commercial preparations of chlorophenoxy herbicides may contain contaminants such as polychlorinated dibenzo- dioxins and furans [2], although the most toxic congener, 2,3,7,8-tetrachlorodibenzo-*p*-dioxin (2,3,7,8-TCDD), a contaminant of 2,4,5-trichlorophenoxy acetic acid, has not been found in commercial 2,4-D [3]. Chlorophenoxy herbicides are transported in the environment for both short and long distances (tens or hundreds of miles) from their point of application [4,5]. 2,4-D has been found in house dust [6]. The presence of 2,4-D has been confirmed in streams and drinking water reservoirs [7,8]. 2,4-D has been classified as a hazardous air pollutant [9] and is included as a regulated contaminant in the National Primary Drinking Water Regulations [10].

Spring wheat, a major field crop in Minnesota, Montana, North Dakota, and South Dakota, was treated during the 1980s and 1990s for at least 85% of its acreage with chlorophenoxy herbicides for control of broadleaf weeds [11]. Several population studies have investigated the potential association between level of spring wheat farming and increased rates of adverse effects in these states. Two studies compared agricultural regions in Minnesota [12,13]. Other studies were based on the combined rural, agricultural counties of Minnesota, Montana, North and South Dakota. Wheat acreage per county was used as a surrogate exposure measure, because information on herbicide use by county was unavailable. The combined population studies showed that more intense wheat farming per county was associated with statistically significant higher rates of birth malformations, and mortality from cancers, acute myocardial infarction, typy-2 diabetes, and renal disease [14-16]. The excess adverse health effects observed in the general population in high-wheat versus low-wheat counties, and the high use of chlorophenoxy herbicides applied to spring wheat, suggested that environmental exposures to these herbicides might be associated with risk factors for these diseases. It is fairly well accepted that a higher prevalence of a disease in one geographic area than in another area may be due to an environmental agent [17].

As an approach toward investigating if environmental exposures to chlorophenoxy herbicides in high spring-wheat counties of Minnesota, Montana, North Dakota, and South Dakota played a role in the excess mortality from acute myocardial infarction and type-2 diabetes, a study was conducted involving subjects that had been tested for presence of urinary 2,4-D, an indicator of a previous recent exposure. This cross-sectional study was based on The National Health and Nutrition Examination Survey III, 1988-1994 (NHANES III), one of several surveys conducted by the Centers for Disease Control and Prevention (CDC) providing national estimates of the health and nutritional status of the non-institutionalized U.S. population. A subset of the NHANES III subjects, 20-59 years of age, was selected to participate in the Priority Toxicant Reference Range Study for the purpose of assessing levels of common pesticides (including 2,4-D) and volatile organic compounds (VOC) in blood or urine [18]. No formal statistical sampling procedure was used to recruit the volunteers. Therefore these volunteers were not representative of the U.S. population. It is not known if their 1988-1994 2,4-D exposure levels were comparable to the levels of residents of spring wheat growing counties in Minnesota, Montana, North Dakota, and South Dakota, who were environmentally exposed to chlorophenoxy herbicides during the spring season, most likely for several consecutive years during the 1980s and 1990s. In the current study, subjects with urinary 2,4-D level above and below the level of detection, were compared. The focus of the investigation was on adverse changes of lipids and glucose metabolism, which have been implicated in the pathogenesis of acute myocardial infarction and type-2 diabetes [19,20]. The starting point for the analyses was based on the following information: 2,4-D, MCPA and the lipid lowering drug clofibrate (2-[4-chlorophenoxy]-2-methylpropionic acid ethyl ester) have similar chemical structures, are lipid lowering in rats, and are PPARα ligands [21,22]; triglycerides and high density lipoprotein cholesterol (HDL) are negatively correlated in humans [23,24].

## Methods

### Data Source and exclusion criteria

NHANES III survey data are publicly available and do not contain any identifiable private information. Therefore any study based on these data is excluded from IRB approval. The purpose of the current study was to investigate effects occurring in healthy subjects soon after an exposure to 2,4-D (half-life of less than one day) as indicated by its presence in the urine. For that reason the following exclusion criteria were used: those that had a history of congestive heart failure, heart attack, diabetes, thyroid disease, lupus, and cancer, were excluded. In addition, subjects with a white blood cell count >12 × 10^9^/L, C-reactive protein >10 mg/dl, or glycosylated hemoglobin (HbA1c) >8%, which could be indications of inflammatory or metabolic problems, were excluded. Urinary levels of 2,4-D were determined by the isotope dilution and tandem mass spectrometry technique [25]. Subjects with urinary 2,4-D levels below the LOD (1 mg/dl) were given the value 0.7071 mg/dl [20].

### Variables and statistical analyses

The analyses included Wilcoxon rank-sum tests, Spearman correlations, and linear regression models [26]. Because only 14% of the values of urinary 2,4-D, the exposure variable of interest, were above the LOD, it was decided to use urinary 2,4-D as a binary variable, above versus below the LOD, with values 1 and 0, respectively. Associations with 2,4-D were investigated for the following natural log-transformed outcome variables: HDL, triglycerides, nonHDL, insulin, C-peptide, plasma glucose, and thyroid stimulating hormone (TSH). The selection of these variables was based on their availability and known association with heart failure and insulin resistance [18,19]. Low density lipoprotein cholesterol (LDL) values were missing for 45% of the subjects. Therefore, the variable nonHDL was created by subtracting values of HDL from values of total cholesterol. This definition of nonHDL includes all cholesterol present in lipoprotein particles considered to be atherogenic [27,28]. The linear regression models included the following explanatory variables: 2,4-D (binary); HDL (continuous, log-transformed, included in all models except where HDL itself was the dependent variable); urinary creatinine (continuous, log-transformed); gender (0 = female, 1 = male); age (continuous); BMI categorized as <25 (referent), 25-29, and ≥ 30; ethnicity categorized as non-Hispanic White (referent), non-Hispanic Black, Mexican American, and "other ethnicity"; smoking categorized as non-smoker (referent), past smoker, and active smoker. Four additional variables were checked for their effect on the beta coefficient for binary 2,4-D using the change-in-estimate method [29]: alcohol consumption categorized as at least 30 drinks a month (wine, beer, or liquor) or less than 30 drinks a month (referent); education categorized as high-school completed or not completed (referent); household income categorized as at least $20,000/year or less than $20,000/year (referent); hours of fasting prior to blood sample (continuous).

Linear regression analyses were run for all available subjects. In addition, two subpopulations that were expected to be more susceptible, were selected: 1) subjects with glycosylated hemoglobin (HbA1c) above the median (5.1%) of the total population, and 2) subjects with thyroxine (T4) at or below the median (8.5 μg/dl) of the total population. T4 and HbA1c with half-lives of 7 and 120 days respectively, are likely to be indicators of health status prior to rather than as a consequence of a recent 2,4-D exposure. High HbA1c and low T4 are known to be associated with cardiac and metabolic adverse effects [18]. Subjects with either one of these conditions may be more vulnerable to potential effects from chlorophenoxy herbicides. HbA1c and T4 could have been included in the model for all available subjects as a continuous or categorical variable. However, it was decided to run the regression analyses separately for these susceptible subpopulations for ease of interpretation. Regression results were presented as estimates with 95% confidence intervals (CI). Because no formal statistical sampling procedure was used to recruit the volunteers, weighting was not recommended for the analyses [20].

## Results

Among the 1338 available subjects that volunteered for the NHANES III VOC study, 375 had missing data on urinary 2,4-D. From the remaining 963 subjects, 236 subjects were excluded based on the exclusion criteria for this study. Among the resulting 727 healthy subjects, 102 (14%) had 2,4-D levels above the LOD ranging from 1 to 28 mg/dl. Table 1 presents characteristics of these subjects based on their 2,4-D status. Subjects exposed to 2,4-D had lower HDL (46.9 versus 51.3 mg/dl, p-value = 0.004), and higher urinary creatinine (201 versus 145 mg/dl, p < 0.0001). No other significant differences among the continuous characteristics were observed.

**Table 1:** Characteristics of NHANES III subjects by urinary 2,4-D below and above the LOD

Characteristics		Urinary 2,4-D	
	
	Missing(n)	< LOD (n = 625)	≥ **LOD (n = 102)**
	
		%	%
Male	0	57	66

Ethnicity	0		

Non-Hispanic White		36	43

Non-Hispanic Black		36	26

Mexican American		25	25

Other		2	5

Smoking	0		

Nonsmokers		49	50

Past smokers		18	16

Active smokers		33	34

Alcohol use ≥ 30 times/month	0	9	7

High school completed	0	71	70

Income ≥ 20 K	6	58	48

BMI (kg/m^2^)	9		

< 25		41	40

25-29		37	37

≥ 30		22	24

			

		**Mean (SE)**	**Mean (SE)**

Age (years)	0	35.6 (0.4)	34.7 (1.1)

Urinary creatinine, mg/dl *	0	144.6 (3.5)	200.5 (9.1)

Hours of fasting	3	11.2 (0.2)	11.8 (0.5)

Thyroxine, μg/dl	10	8.59 (0.08)	8.49 (0.20)

Glycosylated hemoglobin, %	2	5.16 (0.02)	5.17 (0.05)

Outcome variables			

HDL cholesterol, mg/dl *	9	51.3 (0.6)	46.9 (1.3)

Triglycerides, mg/dl	9	127.0 (4.1)	139.0 (10.9)

Non-HDL cholesterol, mg/dl	9	144.4 (1.7)	146.3 (4.5)

Serum insulin, μU/ml	7	10.6 (0.3)	11.3 (0.8)

Serum C-peptide, pmol/ml	8	0.65 (0.02)	0.69 (0.05)

Plasma glucose, mg/dl	6	91.2 (0.5)	91.3 (1.4)

TSH, mU/L	6	1.67 (0.05)	1.57 (0.13)

* Wilcoxon test (2-sided) shows a statistically significant difference between subjects with urinary 2,4-D

above and below the LOD with regard to HDL (p-value = 0.006) and urinary creatinine (p-value < 0.0001).

**Figure 1:**
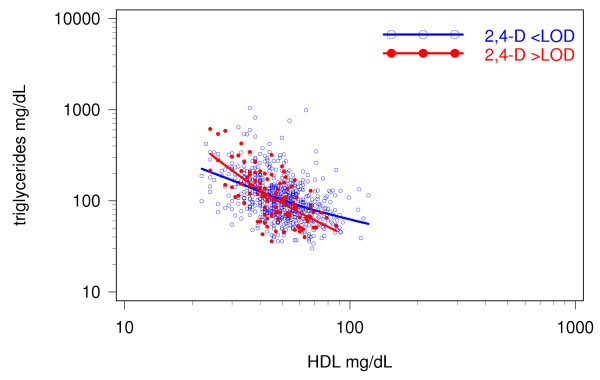
**Effect of 2,4-D on the HDL-triglycerides association**. The graph displays unadjusted data for combined males and females, age 20-59. The negative association between HDL and triglycerides is displayed for subjects with urinary 2,4-D below the level of detection (blue circles and line), and above the level of detection (red dots and line).

Figure 1 shows that the effect of urinary 2,4-D on triglycerides is not additive, as illustrated by the intersection of the two smoothed interpolation lines representing subjects with urinary 2,4-D below and above the level of detection. The triglyceride-HDL Spearman correlation was stronger for the 2,4-D positive subjects (r = -0.61, p-value < 0.0001) than for the 2,4-D negative subjects (r = -0.47, p-value < 0.0001). Subjects with HDL levels of < 40 mg/dl showed increased levels of triglycerides associated with 2,4-D, while subjects with HDL ≥ 40 mg/dl showed a decrease. This association was further explored in a linear regression model including an interaction term log(HDL)*2,4-D and adjusting for potential confounders. Similar regression models were run to evaluate the effect of the log(HDL)*2,4-D interaction on nonHDL, insulin, C-peptide, plasma glucose, and TSH.

To determine which one of the additional four predictor variables should be included in the models based on the change of the 2,4-D coefficient, separate regression analyses (main effects only) were run for each dependent variable including only subjects with low HDL (< 40 mg/dl), which was the more 2,4-D-reactive group (data not shown). It would have been difficult to observe a change-of-effect in a model including all subjects with 2,4-D present both as a main effect and in the interaction term. The results from these simplified models showed that education had no effect, but income, alcohol consumption and hours of fasting did change the estimate of 2,4-D by more than 10% for several of the models. Therefore, all final models included the additional terms for income, alcohol consumption and hours of fasting.

The regression model for HDL (main effects only, Table 2) showed that 2,4-D was associated with a borderline significant 5% decrease of HDL when all available subjects were included (β-coefficient = -0.048, 95% CI [-0.101, 0.006], p-value = 0.08), and a significant 9% decrease for subjects in the lower 50% of T4 (β-coefficient = -0.09, 95% CI [-0.160, -0.016], p-value = 0.02). Other factors associated with a decrease in HDL were being male, active smoking, and BMI ≥ 25. Non-Hispanic Black ethnicity and alcohol consumption were associated with increased levels of HDL.

**Table 2:** Linear regression analysis of log(HDL) on 2,4-D and covariates for ages 20-59, NHANES III, 1988-1994.

Independent variables	All available	Susceptible subpopulations	
		
	subjects (n = 709)	HbA1c > 5.1%	T4 ≤ 8.5 μg/dl
		(n = 345)	(n = 364)
	
	Estimate (95% CI)	Estimate (95% CI)	Estimate (95% CI)
Intercept	4.227	4.107	4.209
	(4.062, 4.391)^a^	(3.871, 4.343)^a^	(3.965, 4.453)^a^

2,4-D, binary	-0.048	-0.015	-0.088
	(-0.101, 0.006)	(-0.090, 0.061)	(-0.160, -0.016)^c^

Log (urinary creatinine)	-0.027	-0.022	-0.032
	(-0.056, 0.001)	(-0.063, 0.019)	(-0.074, 0.010)

Gender	-0.145	-0.100	-0.145
	(-0.184, -0.105)^a^	(-0.157, -0.043)^a^	(-0.203, -0.085)^a^

Age (years)	0.001	0.001	0.001
	(-0.002, 0.002)	(-0.002, 0.003)	(-0.002, 0.003)

Non-Hispanic Black	0.124	0.149	0.154
	(0.078, 0.170)^a^	(0.083, 0.215)^a^	(0.089, 0.219)^a^

Mexican American	-0.032	0.021	-0.017
	(-0.082, 0.017)	(-0.053, 0.094)	(-0.093, 0.060)

Other ethnicity	0.108	0.176	0.232
	(-0.008, 0.223)	(0.025, 0.327)^c^	(0.068, 0.396)^b^

Past smoker	-0.040	-0.011	-0.073
	(-0.092, 0.012)	(-0.082, 0.060)	(-0.147, 0.001)

Active smoker	-0.084	-0.094	-0.097
	(-0.126, -0.041)^a^	(-0.154, -0.034)^b^	(-0.159, -0.035)^b^

BMI 25-29 kg/m^2^	-0.178	-0.201	-0.156
	(-0.220, -0.136)^a^	(-0.262, -0.140)^a^	(-0.216, -0.096)^a^

BMI ≥ 30 kg/m^2^	-0.230	-0.224	-0.230
	(-0.279, -0.181)^a^	(-0.289, -0.160)^a^	(-0.304, -0.157)^a^

Alcohol consumption	0.101	0.069	0.125
	(0.034, 0.168)^b^	(-0.022, 0.159)	(0.039, 0.210)^b^

Income ≥ 20K (%)	-0.022	-0.024	-0.039
	(-0.061, 0.018)	(-0.078, 0.030)	(-0.098, 0.019)

Hours of fasting	-0.001	-0.001	0.002
	(-0.005, 0.004)	(-0.006, 0.006)	(-0.004, 0.008)

^a ^p ≤ 0.001, ^b ^p ≤ 0.01, ^c ^p ≤ 0.05

Main-effects-only regression models for triglycerides, nonHDL, insulin, C-peptide, plasma glucose, and TSH did not show any 2,4-D associated effects (data not shown). However, inclusion of the log(HDL)*2,4-D interaction term identified several significant effects. To facilitate interpretation of this interaction, an equivalent model was used replacing the log(HDL)*2,4-D interaction term with two separate log(HDL) terms, one for subjects with 2,4-D < LOD and one for subjects with 2,4-D ≥ LOD. Results from this second model are presented (Tables 3, 4, 5, 6, 7 and 8), as well as the p-value for the interaction term.

**Table 3:** Linear regression analysis of log(triglycerides) on 2,4-D and covariates for ages 20-59, NHANES III, 1988-1994.

Independent variables	All available subjects (n = 708)	Susceptible subpopulations	
		
		HbA1c > 5.1%	T4 ≤ 8.5 μg/dl
		(n = 345)	(n = 344)
	
	Estimate (95% CI)	Estimate (95% CI)	Estimate (95% CI)
Intercept	6.993	6.575	6.976
	(6.296, 7.690)^a^	(5.525, 7.624)^a^	(5.929, 8.023)^a^

2,4-D, binary	1.804	3.119	1.791
	(0.410, 3.199)^c^	(0.958, 5.279)^b^	(-0.180, 3.761)

Log(HDL) for 2,4-D < LOD	-0.644	-0.570	-0.660
	(-0.791, -0.497)^a^	(-0.798, -0.342)^a^	(-0.879, -0.440)^a^

Log(HDL) for 2,4-D ≥ LOD	-1.119	-1.378	-1.150
	(-1.467, -0.771)^a^	(-1.908, -0.847)^a^	(-1.635, -0.664)^a^

Log (urinary creatinine)	-0.026	-0.001	0.006
	(-0.080, 0.028)	(-0.082, 0.081)	(-0.076, 0.088)

Gender	0.034	0.004	0.006
	(-0.043, 0.112)	(-0.111, 0.120)	(-0.114, 0.126)

Age (years)	0.007	0.008	0.008
	(0.004, 0.011)^a^	(0.003, 0.013)^b^	(0.003, 0.013)^b^

Non-Hispanic Black	-0.130	-0.119	-0.160
	(-0.219, -0.041)^b^	(-0.255, 0.016)	(-0.292, -0.027)^c^

Mexican American	0.079	0.130	0.099
	(-0.014, 0.172)	(-0.016, 0.276)	(-0.053, 0.251)

Other ethnicity	0.185	0.077	0.359
	(-0.033, 0.402)	(-0.226, 0.380)	(0.040, 0.677)^c^

Past smoker	0.019	-0.012	0.040
	(-0.079, 0.117)	(-0.154, 0.130)	(-0.104, 0.183)

Active smoker	0.103	0.028	0.080
	(0.022, 0.183)^b^	(-0.093, 0.148)	(-0.043, 0.202)

BMI 25-29 kg/m^2^	0.242	0.245	0.296
	(0.159, 0.325)^a^	(0.117, 0.373)^a^	(0.173, 0.419)^a^

BMI ≥ 30 kg/m^2^	0.346	0.277	0.315
	(0.248, 0.444)^a^	(0.140, 0.415)^a^	(0.162, 0.468)^a^

Alcohol consumption	0.056	0.094	0.050
	(-0.071, 0.184)	(-0.088, 0.275)	(-0.117, 0.217)

Income ≥ 20K (%)	-0.015	0.045	0.003
	(-0.090, 0.060)	(-0.064, 0.154)	(-0.113, 0.119)

Hours of fasting	-0.014	-0.013	-0.020
	(-0.022, -0.006)^a^	(-0.025, -0.001)^c^	(-0.032, -0.007)^b^

^a ^p ≤ 0.001, ^b ^p ≤ 0.01, ^c ^p ≤ 0.05

P-values for the interaction term log(HDL)*2,4-D are 0.01 for all subjects, 0.005 for subjects with HbA1c > 5.1%, and 0.06 for subjects with T4 ≤ 8.5 μg/dl.

**Table 4:** Linear regression analysis of log(nonHDL) on 2,4-D and covariates for ages 20-59, NHANES III, 1988-1994.

Independent variables	All available subjects (n = 709)	Susceptible subpopulations	
		
		HbA1c > 5.1%	T4 ≤ 8.5 μg/dl
		(n = 345)	(n = 345)
	
	Estimate (95% CI)	Estimate (95% CI)	Estimate (95% CI)
Intercept	5.299	5.239	5.690
	(4.907, 5.691)^a^	(4.660, 5.818)^a^	(5.141, 6.239)^a^

2,4-D, binary	-0.205	-0.187	-0.270
	(-0.989, 0.579)	(-1.379, 1.005)	(-1.303, 0.764)

Log(HDL) for 2,4-D < LOD	-0.191	-0.179	-0.254
	(-0.274, -0.109)^a^	(-0.305, -0.054)^b^	(-0.369, -0.139)^a^

Log(HDL) fo4 2,4-D ≥ LOD	-0.140	-0.124	0.187
	(-0.336, 0.056)	(-0.417, 0.168)	(-0.442, 0.067)

Log (urinary creatinine)	0.001	0.009	-0.035
	(-0.030, 0.031)	(-0.036, 0.054)	(-0.078, 0.008)

Gender	0.046	0.058	0.086
	(0.002, 0.089)^c^	(-0.006, 0.121)	(0.023, 0.149)^b^

Age (years)	0.007	0.008	0.007
	(0.005, 0.009)^a^	(0.005, 0.010)^a^	(0.004, 0.010)^a^

Non-Hispanic Black	-0.058	-0.073	-0.042
	(-0.107, -0.008)^c^	(-0.148, 0.002)	(-0.111, 0.027)

Mexican American	0.032	0.028	0.041
	(-0.021, 0.084)	(-0.053, 0.108)	(-0.038, 0.120)

Other ethnicity	-0.002	-0.067	0.014
	(-0.124, 0.121)	(-0.234, 0.100)	(-0.154, 0.181)

Past smoker	0.043	0.041	0.026
	(-0.012, 0.098)	(-0.037, 0.120)	(-0.049, 0.101)

Active smoker	0.043	0.022	0.023
	(-0.003, 0.088)	(-0.045, 0.088)	(-0.041, 0.088)

BMI 25-29 kg/m^2^	0.128	0.084	0.148
	(0.081, 0.174)^a^	(0.013, 0.155)^c^	(0.084, 0.213)^a^

BMI ≥ 30 kg/m^2^	0.218	0.153	0.212
	(0.163, 0.273)^a^	(0.77, 0.229)^a^	(0.132, 0.292)^a^

Alcohol consumption	0.014	0.019	-0.020
	(-0.058, 0.085)	(-0.81), 0.120)	(-0.107, 0.068)

Income ≥ 20K (%)	0.053	0.057	0.047
	(0.011, 0.095)^c^	(-0.003, 0.117)	(-0.014, 0.107)

Hours of fasting	-0.003	-0.002	-0.004
	(-0.007, 0.002)	(-0.008, 0.005)	(-0.011, 0.002)

^a ^p ≤ 0.001, ^b ^p ≤ 0.01, ^c ^p ≤ 0.05

P-values for the interaction term log(HDL)*2,4-D are 0.62 for all subjects, 0.73 for subjects with HbA1c > 5.1%, and 0.63 for subjects with T4 ≤ 8.5 μg/dl.

**Table 5:** Linear regression analysis of log(insulin) on 2,4-D and covariates for ages 20-59, NHANES III, 1988-1994.

Independent variables	All available subjects (n = 703)	Susceptible subpopulations	
		
		HbA1c > 5.1%	T4 ≤ 8.5 μg/dl
		(n = 340)	(n = 342)
	
	Estimate (95% CI)	Estimate (95% CI)	Estimate (95% CI)
Intercept	3.669	3.495	2.962
	(2.973, 4.364)^a^	(2.430, 4,560)^a^	(1.944, 3.980)^a^

2,4-D, binary	0.763	2.611	1.451
	(-0.616, 2.142)	(0.447, 4.775)^c^	(-0.442, 3.344)

Log(HDL) for 2,4-D < LOD	-0.412	-0.396	-0.295
	(-0.558, -0.265)^a^	(-0.627, -0.165)^a^	(-0.508, -0.081)^b^

Log(HDL) for 2,4-D ≥ 2,4-D	-0.600	-1.076	-0.657
	(-0.944, -0.257)^a^	(-1.605, -0.547)^c^	(-1.123, -0.191)^b^

Log(urinary creatinine)	-0.009	0.015	0.001
	(-0.063, 0.044)	(-0.067, 0.098)	(-0.078, 0.080)

Gender	-0.093	-0.087	-0.044
	(-0.170, -0.016)^c^	(-0.203, 0.030)	(-0.160, 0.071)

Age (years)	0.001	0.002	0.002
	(-0.003, 0.004)	(-0.004, 0.007)	(-0.003, 0.007)

Non-Hispanic Black	0.171	0.183	0.175
	(0.082, 0.259)^a^	(0.047, 0.319)^b^	(0.046, 0.303)^b^

Mexican American	0.096	0.087	0.129
	(0.004, 0.188)^c^	(-0.059, 0.234)	(-0.017, 0.274)

Other ethnicity	0.232	0.074	0.131
	(0.017, 0.447)^c^	(-0.228, 0.376)	(-0.175, 0.437)

Past smoker	-0.023	-0.046	0.031
	(-0.120, 0.074)	(-0.188, 0.096)	(-0.107, 0.168)

Active smoker	-0.112	-0.186	-0.064
	(-0.192, -0.033)^b^	(-0.307, -0.066)^b^	(-0.182, 0.054)

BMI 25-29 kg/m^2^	0.310	0.325	0.368
	(0.228, 0.392)^a^	(0.197, 0.453)^a^	(0.249, 0.486)^a^

BMI ≥ 30 kg/m^2^	0.687	0.672	0.795
	(0.590, 0.785)^a^	(0.533, 0.811)^a^	(0.648, 0.943)^a^

Alcohol consumption	-0.081	-0.089	-0.052
	(-0.209, 0.046)	(-0.275, 0.097)	(-0.213, 0.109)

Income ≥ 20K (%)	-0.045	-0.013	0.004
	(-0.119, 0.030)	(-0.123, 0.096)	(-0.108, 0.116)

Hours of fasting	-0.008	-0.007	-0.010
	(-0.016, 0.000)^c^	(-0.018, 0.005)	(-0.021, 0.002)

^a ^p ≤ 0.001, ^b ^p ≤ 0.01, ^c ^p ≤ 0.05

P-values for the interaction term log(HDL)*2,4-D are 0.30 for all subjects, 0.02 for subjects with HbA1c > 5.1%, and 0.15 for subjects with T4 ≤ 8.5 μg/dl.

**Table 6:** Linear regression analysis of log(C-peptide) on 2,4-D and covariates for ages 20-59, NHANES III, 1988-1994.

Independent variables	All available subjects (n = 706)	Susceptible subpopulations	
		
		HbA1c > 5.1%	T4 ≤ 8.5 μg/dl
		(n = 343)	(n = 343)
	
	Estimate (95% CI)	Estimate (95% CI)	Estimate (95% CI)
Intercept	1.258	0.707	0.778
	(0.294, 2.221)^b^	(-0.585, 1.999)	(-0.733, 2.289)

2,4-D, binary	1.567	3.599	1.733
	(-0.344, 3.478)	(0.970, 6.228)^b^	(-1.078, 4.545)

Log(HDL) for 2,4-D < LOD	-0.562	-0.438	-0.558
	(-0.765, -0.358)^a^	(-0.719, -0.157)^b^	(-0.875, -0.241)^a^

Log(HDL) for 2,4-D ≥ LOD	-0.986	-1.397	-1.029
	(-1.462, -0.509)^a^	(-2.040, -0.754)^a^	(-1.721, -0.336)^b^

Log(urinary creatinine)	-0.013	0.018	0.029
	(-0.087, 0.062)	(-0.081, 0.116)	(-0.088, 0.147)

Gender	-0.191	-0.184	-0.167
	(-0.297, -0.086)^a^	(-0.324, -0.044)^b^	(-0.337, 0.004)

Age (years)	0.005	0.005	0.009
	(0.000, 0.010)^c^	(-0.002, 0.011)	(0.001, 0.017)^c^

Non-Hispanic Black	-0.038	-0.045	0.025
	(-0.161, 0.084)	(-0.209, 0.119)	(-0.166, 0.216)

Mexican American	0.034	0.016	0.100
	(-0.093, 0.162)	(-0.161, 0.193)	(-0.117, 0.316)

Other ethnicity	0.151	0.041	0.151
	(-0.147, 0.449)	(-0.326, 0.408)	(-0.303, 0.605)

Past smoker	-0.022	0.001	-0.048
	(-0.156, 0.112)	(-0.170, 0.173)	(-0.253. 0.156)

Active smoker	0.002	-0.043	-0.059
	(-0.108, 0.112)	(-0.189, 0.104)	(-0.235, 0.117)

BMI 25-29 kg/m^2^	0.480	0.480	0.607
	(0.366, 0.594)^a^	(0.325, 0.636)^a^	(0.431, 0.783)^a^

BMI ≥ 30 kg/m^2^	0.890	0.858	1.067
	(0.756, 1.024)^a^	(0.691, 1.025)^a^	(0.849, 1.286)^a^

Alcohol consumption	-0.015	-0.022	0.092
	(-0.192, 0.162)	(-0.248, 0.205)	(-0.148, 0.331)

Income ≥ 20K (%)	-0.052	0.022	0.008
	(-0.155, 0.050)	(-0.110, 0.154)	(-0.157, 0.174)

Hours of fasting	-0.008	-0.008	-0.015
	(-0.019, 0.004)	(-0.022, 0.007)	(-0.033, 0.002)

^a ^p ≤ 0.001, ^b ^p ≤ 0.01, ^c ^p ≤ 0.05

P-values for the interaction term log(HDL)*2,4-D are 0.10 for all subjects, 0.006 for subjects with HbA1c > 5.1%, and 0.21 for subjects with T4 ≤ 8.5 μg/dl.

**Table 7:** Linear regression analysis of log(plasma glucose) on 2,4-D and covariates for ages 20-59, NHANES III, 1988-1994.

Independent variables	All available subjects (n = 708)	Susceptible subpopulations	
		
		HbA1c > 5.1%	T4 ≤ 8.5 μg/dl
		(n = 344)	(n = 344)
	
	Estimate (95% CI)	Estimate (95% CI)	Estimate (95% CI)
Intercept	4.490	4.559	4.605
	(4.340, 4.639)^a^	(4.316, 4.801)^a^	(4.381, 4.829)^a^

2,4-D, binary	0.009	0.175	0.059
	(-0.290, 0.308)	(-0.323, 0.674)	(-0.364, 0.481)

Log(HDL) for 2,4-D < LOD	-0.025	-0.037	-0.044
	(-0.056, 0.007)	(-0.090, 0.016)	(-0.091, 0.003)

Log(HDL) for 2,4-D ≥ LOD	-0.027	-0.080	-0.061
	(-0.102, 0.047)	(-0.202, 0.042)	(-0.165, 0.043)

Log(urinary creatinine)	-0.003	-0.010	-0.010
	(-0.014, 0.009)	(-0.028, 0.009)	(-0.028, 0.007)

Gender	0.030	0.022	0.032
	(0.014, 0.047)^a^	(-0.005, 0.049)	(0.006, 0.057)^c^

Age (years)	0.003	0.003	0.002
	(0.002, 0.003)^a^	(0.002, 0.004)^a^	(0.001, 0.003)^a^

Non-Hispanic Black	-0.017	-0.007	-0.004
	(-0.036, 0.002)	(-0.038, 0.025)	(-0.033, 0.024)

Mexican American	0.006	0.008	0.004
	(-0.014, 0.025)	(-0.026, 0.042)	(-0.028, 0.037)

Other ethnicity	0.063	0.046	0.084
	(0.016, 0.109)^b^	(-0.023, 0.116)	(0.016, 0.152)^c^

Past smoker	0.001	-0.011	-0.008
	(-0.021, 0.022)	(-0.043, 0.022)	(-0.039, 0.023)

Active smoker	-0.005	-0.016	-0.004
	(-0.023, 0.012)	(-0.044, 0.012)	(-0.030, 0.023)

BMI 25-29 kg/m^2^	0.024	0.021	0.048
	(0.006, 0.042)^b^	(-0.009, 0.050)	(0.021, 0.074)^a^

BMI ≥ 30 kg/m^2^	0.070	0.066	0.097
	(0.049, 0.091)^a^	(0.034, 0.097)^a^	(0.064, 0.129)^a^

Alcohol consumption	-0.019	-0.040	-0.013
	(-0.046, 0.008)	(-0.082, 0.002)	(-0.049, 0.023)

Income ≥ 20K (%)	-0.007	0.004	-0.001
	(-0.022, 0.010)	(-0.021, 0.029)	(-0.025, 0.024)

Hours of fasting	0.003	0.004	0.001
	(0.001, 0.004)^b^	(0.001, 0.006)^b^	(-0.002, 0.003)

^a ^p ≤ 0.001, ^b ^p ≤ 0.01, ^c ^p ≤ 0.05

P-values for the interaction term log(HDL)*2,4-D are 0.95 for all subjects, 0.52 for subjects with HbA1c > 5.1%, and 0.77 for subjects with T4 ≤ 8.5 μg/dl.

**Table 8:** Linear regression analysis of log(TSH) on 2,4-D and covariates for ages 20-59, NHANES III, 1988-1994.

Independent variables	All available subjects (n = 705)	Susceptible subpopulations	
		
		HbA1c > 5.1%	T4 ≤ 8.5 μg/dl
		(n = 343)	(n = 342)
	
	Estimate (95% CI)	Estimate (95% CI)	Estimate (95% CI)
Intercept	-0.487	0.134	-1.263
	(-1.380, 0.407)	(-1.235, 1.503)	(-2.587, 0.061)

2,4-D, binary	1.380	-0.915	2.537^c^
	(-0.395, 3.154)	(-3.689, 1.858)	(0.066, 5.007)^c^

Log(HDL) for 2,4-D < LOD	0.209	0.125	0.309
	(0.021, 0.397)	(-0.172, 0.422)	(0.032, 0.585)^c^

Log(HDL) for 2,4-D ≥ LOD	-0.177	0.360	-0.371
	(-0.620, 0.266)	(-0.321, 1.042)	(-0.981, 0.239)

Log(urinary creatinine)	-0.010	-0.029	0.067
	(-0.079, 0.059)	(-0.135, 0.076)	(-0.036, 0.170)

Gender	0.024	0.052	0.004
	(-0.075, 0.123)	(-0.097, 0.202)	(-0.147, 0.155)

Age (years)	0.004	0.002	0.007
	(-0.000, 0.009)	(-0.005, 0.009)	(0.000, 0.014)^c^

Non-Hispanic Black	-0.264	-0.312	-0.346
	(-0.377, -0.151)^a^	(-0.486, -0.138)^a^	(-0.512, -0.180)^a^

Mexican American	-0.126	-0.227	-0.167
	(-0.244, -0.007)^c^	(-0.415, -0.040)^c^	(-0.357, 0.023)

Other ethnicity	-0.046	-0.072	-0.111
	(-0.331, 0.238)	(-0.477, 0.334)	(-0.527, 0.306)

Past smoker	-0.049	-0.192	-0.076
	(-0.174, 0.076)	(-0.374, -0.010)^c^	(-0.256, 0.104)

Active smoker	-0.117	-0.260	-0.080
	(-0.220, -0.014)^c^	(-0.416, -0.103)^b^	(-0.235, 0.075)

BMI 25-29 kg/m^2^	0.100	0.106	0.215
	(-0.005, 0.206)	(-0.059, 0.271)	(0.061, 0.369)^b^

BMI ≥ 30 kg/m^2^	0.148	0.156	0.304
	(0.023, 0.274)^c^	(-0.024, 0.336)	(0.111, 0.498)^b^

Alcohol consumption	-0.005	0.032	-0.003
	(-0.167, 0.157)	(-0.202, 0.263)	(-0.212, 0.207)

Income ≥ 20K (%)	-0.036	-0.053	0.015
	(-0.132, 0.059)	(-0.193, 0.087)	(-0.130, 0.161)

Hours of fasting	0.002	-0.001	-0.011
	(-0.009, 0.012)	(-0.016, 0.015)	(-0.026, 0.005)

^a ^p ≤ 0.001, ^b ^p ≤ 0.01, ^c ^p ≤ 0.05

P-values for the interaction term log(HDL)*2,4-D are 0.10 for all subjects, 0.52 for subjects with HbA1c > 5.1%, and 0.04 for subjects with T4 ≤ 8.5 μg/dl.

Results of the regression model for triglycerides showed that both urinary 2,4-D and HDL played a role (Table 3). The 2,4-D effect on triglycerides was HDL dependent, as was also observed in Figure 1. The following numeric example illustrates the HDL-associated 2,4-D effect on triglycerides. Using estimates for all available subjects, and ignoring all other explanatory variables (Table 3), it was shown that by solving the equation: "6.993 - 0.644*log(HDL) = 6.993 + 1.804 - 1.119*log(HDL)", 2,4-D had no effect on triglyceride levels at HDL = 48 mg/dl, which agrees with the approximate point of intersection of the two fitted lines in Figure 1. Similar calculations showed that at low HDL, e.g. 25 mg/dl, estimated levels for triglycerides were 137 and 180 mg/dl for 2,4-D < and ≥ LOD, respectively. For high HDL, e.g. 60 mg/dl, estimated levels for triglycerides were 78 and 68 mg/dl for 2,4-D < and ≥ LOD, respectively. In other words, 2,4-D was associated with an increase of triglycerides at low HDL, and a decrease at high HDL. Effects were somewhat stronger for the susceptible subgroup with HbA1c levels over 5.1% as indicated by the non-overlapping confidence intervals for the two log(HDL) terms, and the p-value of 0.005 for the log(HDL)*2,4-D interaction term, in comparison to the all-available-subjects model with overlapping confidence intervals and the p-value of 0.01 for the interaction term. Other factors associated with increased levels of triglycerides were age, active smoking, and high BMI. Being non-Hispanic Black and increased hours of fasting were associated with decreased levels of triglycerides.

No association between nonHDL and 2,4-D was observed (Table 4). In the model for all available subjects, male gender, age, high BMI, and income were associated with increased nonHDL. Non-Hispanic Black ethnicity and HDL among subjects with urinary 2,4-D < LOD were associated with a decrease. The effects of 2,4-D on insulin and C-peptide (Tables 5-6) were most pronounced for the susceptible subpopulation with HbA1c over 5.1%. Similar to the numerical triglyceride example, it was shown that among subjects in this susceptible subpopulation with low HDL (25 mg/dl), 2,4-D was associated with an increase in insulin, from 9.2 μU/ml for 2,4-D < LOD to 14.0 μU/ml for 2,4-D ≥ LOD. At high HDL (60 mg/dl) 2,4-D was associated with a slight decrease of insulin, from 6.5 μU/ml for 2,4-D < LOD to 5.5 μU/ml for 2,4-D ≥ LOD. One subject with a value of 102 μU/ml insulin was considered an outlier (the next highest value was 50 μU/ml) and was excluded from the regression analysis. Other adverse factors associated with increased insulin for this susceptible subpopulation were non-Hispanic Black ethnicity and high BMI. Active smoking was protective. Effects on C-peptide were similar to those of insulin. At low HDL (25 mg/dl) 2,4-D was associated with an increase in C-peptide, from 0.50 pmol/ml for 2,4-D < LOD to 0.83 pmol/ml for 2,4-D ≥ LOD. At high HDL (60 mg/dl) 2,4-D was associated with a decrease of C-peptide, from 0.34 pmol/ml for 2,4-D < LOD to 0.24 pmol/ml for 2,4-D ≥ LOD. The two confidence intervals for the log(HDL) terms did not overlap, which was in line with the highly significant p-value of 0.006 for the log(HDL)*2,4-D interaction term. High BMI was an adverse factor for C-peptide. Male gender was protective for this susceptible subpopulation.

Plasma glucose was not associated with 2,4-D (Table 7). Male gender, age, "other" ethnicity, high BMI, and hours of fasting were associated with increased plasma glucose. The association between 2,4-D and TSH (Table 8) was most pronounced for subjects in the susceptible subpopulation with T4 ≤ 8.5 μg/dl. The level of the effect depended on HDL, and followed a similar pattern as for triglycerides, C-peptide, and insulin. Low HDL (25 mg/dl) was associated with an increase in TSH from 0.77 for 2,4-D < LOD to1.08 mU/L for 2,4-D ≥ LOD. High HDL (60 mg/dl) was associated with a decrease for TSH from 1.00 mU/L for 2,4-D < LOD to 0.78 mU/L for 2,4-D ≥ LOD. The confidence intervals for the two log(HDL) terms overlapped, in line with the (not highly) significant p-value of 0.04 for the interaction term. Age and BMI were associated with increased levels of TSH, while non-Hispanic Black ethnicity was protective in the low T4 model. Among the 727 subjects available for the analyses, 94 subjects (13.4%) had TSH > 2.5 mU/L, and T4 ≥ 4.5 μg/dl, which may indicate early or mild thyroid dysfunction [27]. Four subjects (0.6%) had TSH > 2.5 mU/L, T4 < 4.5 μg/dl, which could be defined as having overt hypothyroidism based on the same definition.

## Discussion

The main findings associated with presence of urinary 2,4-D in healthy NHANES III subjects are the overall 5-9% decrease of HDL, and the HDL-dependent effects on triglycerides, insulin, C-peptide, and TSH, especially in the more susceptible subpopulations. Inclusion of the interaction term "log(HDL)*2,4-D" in the regression model or using an equivalent model with separate terms for log(HDL) with 2,4-D < LOD and for log(HDL) with 2,4-D ≥ LOD, showed that subjects with low HDL experienced higher rates of 2,4-D-associated adverse effects than subjects with high HDL. Without this interaction term or its equivalent in the regression model, the 2.4-D effect would have been completely missed. Previous exposures to pollutants, which may or may not have included 2,4-D, may have contributed to the susceptibility of subjects, by lowering T4 or HDL, or increasing HbA1c. The effect of increased susceptibility due to existing conditions such as having high HbA1c or low T4, was tested in the regression analyses. The effect of low HDL as an existing condition prior to 2,4-D exposure could not be tested in this cross-sectional study. Only a follow-up study can determine if a 2,4-D exposure is associated with a further decrease of HDL among low-HDL subjects. The 2.4-D associated adverse effects of decreased HDL, increased triglycerides, insulin, C-peptide, and TSH, may be part of a causal path that eventually may lead to weight gain, acute myocardial infarction, type-2 diabetes, and possibly other diseases. This concept is supported by a recent publication discussing a new model for environmental disease in which previous cumulative effects of different exposures may lead to increased vulnerability, thereby creating favorable conditions for overt disease [30]. It is not clear at this point why no 2,4-D effect was observed for nonHDL in the regression analysis. The lack of an association with plasma glucose may indicate that in this study insulin and C-peptide are the better biomarkers to observe changes in glucose metabolism.

The fact that the estimates for other predictors such as BMI and smoking, are in line with what is known in the literature, supports the credibility of the methods used in this study. For example, both smoking and high BMI are known to be associated with low HDL levels [31]; moderate alcohol consumption is known to increase HDL levels [32,33]. In the current study a decrease of HDL was observed in association with BMI (in the form of a dose response) and with active smoking, while alcohol consumption (30 or more drinks per month) was associated with an increase of HDL (Table 2). In general, BMI was associated with each dependent variable in a dose-response fashion.

The 2,4-D associated adverse effects have been observed in other studies. 2,4-D is known to interfere with thyroid hormone transport and to possibly reduce their levels [34,35]. Thyroid hormones regulate lipid metabolism by controlling the action of key enzymes in the reverse cholesterol transport, in which HDL plays a major role [36-38]. A disturbance of the thyroid hormone homeostasis may impair the reverse cholesterol transport process, which in turn may result in a decrease of HDL, a characteristic of subclinical hypothyroidism [36]. When subclinical hypothyroidism, representing mild thyroid failure, progresses to severe hypothyroidism however, normal or increased HDL levels may be the result [36]. Subclinical hypothyroidism is a known risk factor for lipid abnormalities, endothelial dysfunction, and coronary heart disease [27,36,39,40]. A decrease in HDL plasma levels is associated with an increase in coronary heart disease [41]. This has been observed at all levels of LDL and triglycerides, for both diabetics and nondiabetics, and for both sexes [42-47]. Low HDL is often accompanied by insulin resistance, obesity, and hypertension, which are risk factors for atherosclerosis and are components of the metabolic syndrome [23,43].

The "favorable" triglyceride-lowering effect observed among subjects with high HDL may be similar to the known triglyceride-lowering effect in humans treated with clofibrate or other fibrates with a chlorophenoxy structure such as fenofibrate (1-methylethyl2-[4-(4-chlorobenzoyl) phenoxy]-2-methyl-propanoate) [41]. This is supported by an animal study showing that rats treated with either chlorophenoxy herbicides or clofibrate showed a triglyceride lowering effect [22]. 2,4-D, MCPA, clofibrate, and fenofibrate are synthetic ligands of the peroxisome proliferator receptors PPARα, which activate genes involved in lipid metabolism. This activation results in altered (increased or repressed) transcription of genes encoding for proteins that control lipoprotein metabolism [22,48-51]. PPARs and thyroid hormone receptors belong to the same superfamily of nuclear hormone ligand-activated transcription factors [52]. Adverse effects have been shown in association with clofibrate [48,53,54]. An animal study showed that plasma concentrations of T4 and T3 were decreased in pigs treated for 28 days with clofibrate. This effect, also noted in rats, was thought to be due to increased hepatic glucuronidation of thyroid hormones [55]. The similarities of 2,4-D with pharmaceutical fibrates support the notion that 2,4-D itself may be associated with adverse effects, although contribution by its contaminants can not be ruled out.

### Limitations

A major limitation is the fact that the results apply to this study's subjects only, and are not representative of the U.S. population. In addition, cross-sectional data are not always able to establish cause-and-effect. If the effect takes place immediately after the cause, or is based on a stable system, use of cross-sectional data is appropriate [56]. However, when the system can be defined as dynamic, where the cause produces effects over time, studies using cross-sectional data need to consider reverse causality [57]. Regarding this study, reverse causality would ask if presence of urinary 2,4-D could be considered a consequence of having low HDL. It seems more likely that the observed changes in biomarkers are a consequence of exposure to 2,4-D and/or contaminants. Another limitation exists when exposures to different pollutants taking place at different times are associated with the same adverse effect. It is difficult to separate the contribution by different exposures to the same adverse effect.

## Conclusions

By showing that 2,4-D exposure was associated with adverse changes in lipid levels, glucose metabolism, and TSH levels, which may predispose to heart disease and diabetes, the current study provides support to results from a previous population study that showed excess mortality from these diseases in high-wheat counties [16]. The two types of studies complement each other. The biomarker study contributed information on the toxicity pattern of 2,4-D. The population study contributed information on diseases associated with environmental exposure to chlorophenoxy herbicides. These combined studies are an example of the recommended methodology for studies on health effects associated with environmental exposures [58]. The current study has highlighted the major role of HDL in response to an exposure of 2,4-D. Future studies need to confirm these results, and should further investigate the effect of other environmental pollutants on HDL and the HDL-associated enzyme paraoxonase (PON), a known predictor of coronary events [42].

## Abbreviations

BMI: Body mass index; 2,4-D: 2,4-dichlorophenoxy acetic acid; HbA1c: glycosylated hemoglobin; HDL: high-density lipoprotein cholesterol; CI: confidence interval; LDL: low-density lipoprotein cholesterol; LOD: level of detection; MCPA: 4-methyl-2-chlorophenoxyacetic acid; NHANES III: National Health and Nutrition Examination Survey 1988-1994; PPAR: peroxisome proliferator-activated receptor; T4: thyroxine; TSH: thyroid stimulating hormone; VOC: volatile organic compounds.

## Competing interests

The author declares that they have no competing interests.

## Authors' contributions

The author conducted the literature review, did the analyses, wrote the manuscript, and constructed the tables and figure.
